# The Evolving Landscape of Immunotherapy in Uterine Cancer: A Comprehensive Review

**DOI:** 10.3390/life13071502

**Published:** 2023-07-03

**Authors:** Bashar Haj Hamoud, Romina Marina Sima, Ileana Adela Vacaroiu, Mihai-Teodor Georgescu, Anca Bobirca, Alexandra Gaube, Florin Bobirca, Dragos-Eugen Georgescu

**Affiliations:** 1 Department for Gynecology, Obstetrics and Reproductive Medicine, Saarland University Hospital, 66421 Homburg, Germany; 2 “Bucur Maternity” Obstetrics and Gynecology Discipline, “Carol Davila” University of Medicine and Pharmacy, 020021 Bucharest, Romania; 3 “Sfantul Ioan” Emergency Hospital Nephrology Discipline, “Carol Davila” University of Medicine and Pharmacy, 020021 Bucharest, Romania; 4 “Prof. Dr. Al. Trestioreanu” Oncology Discipline, “Carol Davila” University of Medicine and Pharmacy, 020021 Bucharest, Romania; 5 “Dr. I. Cantacuzino” Clinical Hospital Internal Medicine and Rheumatology Discipline, “Carol Davila” University of Medicine and Pharmacy, 020021 Bucharest, Romania; 6 “Matei Bals” Institute of Infectious Diseases, 021105 Bucharest, Romania; 7 “Dr. Ion Cantacuzino” Surgery Discipline, “Carol Davila” University of Medicine and Pharmacy, 020021 Bucharest, Romania

**Keywords:** immunotherapy, endometrial cancer, tumor microenvironment

## Abstract

Endometrial cancer affects the uterus and is becoming increasingly common and deadly. Although surgery and adjuvant pelvic radiotherapy can often cure the disease when it is contained in the uterus, patients with metastatic or recurrent disease have limited response rates to chemotherapy, targeted agents, and hormonal therapy. To address this unmet clinical need, innovative treatment strategies are needed, and a growing focus on the immunomodulation of the tumor microenvironment has arisen. Current data suggest that active and/or passive immunotherapy may be promising for the treatment of endometrial cancer.

## 1. Introduction

Immunotherapy for uterine (endometrial) cancer is a new field of investigation and treatment, particularly for individuals with advanced disease. Endometrial cancer, often known as uterine cancer, develops when the cells lining the interior of the uterus begin to grow uncontrollably. Cancer can occur in either the upper region (uterine corpus), also known as uterine cancer, or the lower region (uterine cervix), also known as cervical cancer. Obesity, hormonal changes in estrogen production, aging, high-fat diets, diabetes, a family history of certain malignancies, and a history of breast or ovarian cancer are all risk factors for uterine cancer. In the case of uterine malignancies, the National Comprehensive Cancer Network advises universal mismatch repair protein testing or microsatellite instability (MSI) testing. Uterine cancer is the sixth most prevalent kind of cancer in women and the fifteenth most common cancer type in the general population. Every year, around 382,000 new cases are diagnosed globally, with an estimated 90,000 fatalities. The United States is responsible for around 66,000 of these new cases and 13,000 fatalities. The prognosis of endometrial cancer is mostly dictated by disease stage, grade, and histology. Individuals diagnosed with stage 1 illness have a five-year survival rate of 80 to 90 percent, those identified with stage 2 have a rate of 70 to 80 percent, and those diagnosed with stages 3 and 4 have a rate of 20 to 60 percent. Endometrial cancer (EC) is a prevalent malignancy in women and the most common gynecologic cancer in industrialized nations, with an estimated 63,230 new cases in the United States in 2018 [1]. Factors such as early menarche, late menopause, obesity, tamoxifen exposure, and nulliparity have been associated with an increase in circulating estrogen levels, which can contribute to the development of EC. While most EC cases are sporadic, 2–5% are familial and can be linked to specific germline mutations in mismatch repair genes [2]. Genetic testing is becoming increasingly important in the diagnosis and management of EC, including the identification of several clusters of genetic mutations and polymorphisms and the investigation of intracellular signaling system modifications in carcinogenesis [3]. Currently, for locally advanced cancer, surgery is the main therapeutic option, and the assessment is carried out through a pathologic investigation. If postoperative adverse factors are present, then adjuvant treatment is recommended, such as external beam radiation therapy and brachytherapy. Adequate dose coverage is obtained if the position of the uterus is considered and imaging is used [4,5].

While the previous generation of multimodal treatment options achieved full responses in advanced gynecological cancers [6], new targeted therapy options have been developed and successfully implemented in recent years. This development was possible as a result of the designation of various clusters of genetic mutations and polymorphisms; the study of intracellular signaling pathways, modifications in carcinogenesis, and the tumor microenvironment; and the identification of key molecules for the regulation of tumor growth. The immunotherapeutic alternatives for EC currently on the market are discussed in this review, together with the findings of clinical trials and continuing investigations on targeted treatment. Uterine cancer is easily treated if discovered early, but novel treatment options are required for patients with advanced cases and for those who want to avoid surgery and maintain the health of their reproductive systems.

## 2. Discussion 

### 2.1. Genetic and Biological Background 

Traditionally, endometrial cancer (EC) has been categorized into two types based on Bokhman’s concept [7]. Type I is the most common, accounting for 60–70% of cases, and includes endometrioid cancer with a grading between 1 and 2 and the strong expression of hormonal receptors. Type II includes high-grade endometrioid cancer and other histological types such as serous or clear-cell carcinoma. These cancers are typically negative for hormone receptors and have a worse prognosis. In Type I EC, mutations in PTEN are found in 52–78% of lesions, while KRAS mutations are found in 15–43% of cases. Other genes commonly altered include ARID1A and B-catenin, with these mutations typically occurring in the PTEN/PI3K/AKT/mammalian target of the rapamycin (mTOR) pathway. Microsatellite instability (MSI) is present in one-third of Type I EC, while TP53 is mutated in the majority of subtype II cancers [8]. 

The classification of uterine cancer based on research by the Cancer Genome Atlas (TCGA) unveiled distinct molecular subtypes that contribute to our understanding of the disease’s heterogeneity. TCGA identified four main molecular subtypes of uterine cancer: the endometrial hypermutated (POLE-ultramutated), microsatellite instability hypermutated, copy-number low, and copy-number high subtypes [9]. The endometrial hypermutated subtype is characterized by a high mutational burden, primarily driven by POLE exonuclease domain mutations [9]. The microsatellite instability hypermutated subtype is associated with defects in DNA mismatch repair mechanisms, leading to frequent mutations in microsatellite regions [9]. The copy-number low subtype exhibits a relatively stable genome with fewer copy-number alterations, while the copy-number high subtype displays extensive genomic instability and frequent amplifications and deletions [9]. Understanding the molecular subtypes defined by TCGA provides valuable insights into the underlying biology and potential therapeutic strategies tailored to specific subsets of uterine cancer.

The MLH1, MSH2, MSH6, and PMS2 genes, which are involved in the mismatch repair mechanism, are altered in both sporadic and hereditary EC, leading to the microsatellite instability hypermutated subtype (MSI-H), which has a high mutation rate.

Most endometrioid cancers have low copy numbers and low mutation rates, and they commonly have mutations in the genes PTEN, CTNNB1, PIK3CA, ARID1A, and KRAS. This subtype appears to have a similar etiology to colorectal cancers.

High copy-number cancers, such as serous tumors, account for 25% of high-grade endometrioid tumors and are characterized by high copy-number variability, a low mutation rate, frequent TP53 mutations, and poor estrogen and progesterone receptor expression. This subgroup resembles serous ovarian carcinoma and basal-like breast cancer in terms of its characteristics.

A reclassification of EC was made possible by this genetic profile, which may also affect prognosis and help clinicians decide which treatments to administer. Unfortunately, broader clinical applications could not be implemented with the genome sequencing techniques used for TCGA’s project.

More recently, the same molecular groupings have been identified using therapeutically useful techniques, which are less complicated and costly. These cutting-edge technologies, which are based on next-generation sequencing (NGS) techniques, consider important epitopes that can be assessed using very straightforward molecular techniques. This information adds prognostic data and directly affects clinical therapy [10,11].

### 2.2. Identification of the Tumor Microenvironment

Characterizing the immune system components inside the TME is a prerequisite for using the immune system as a cancer treatment.

Given the variations in immune cell makeup across malignancies of the same kind as well as between cancer types, this has proven to be a challenging task. This diversity is a result of immune cells’ ability to perform a variety of activities within the immune system’s main goals of protecting the host and maintaining tissue homeostasis. The phenotypic and functional flexibility of immune cells allows them to perform these jobs. Immune cells play a dual role in supporting host defense and preventing collateral tissue damage in both innate and adaptive immunity. As is widely known, the microenvironment may control the phenotype and function of differentiated myeloid or lymphoid cells at the level of their progenitors throughout their lineage-specific differentiation and after they have grown into fully differentiated cell types. Because of their flexibility, differentiated hematopoietic cells can be thought of as a dynamic continuum rather than as discrete subcategories, which is in line with current thinking on the subject. The variations in the immune cell composition of the endometrium brought on by hormonal factors may help to explain, in part, why constructing a comprehensive picture of endometrial cancer at the molecular level has been so difficult. According to two recent evaluations, the immune system in the endometrium faces a particularly difficult challenge. It must be capable of offering protection from sexually transmitted diseases while also being accommodating enough to permit the growth of an allogeneic fetus. As a result, sex hormones precisely control the immunological activity at this location in the female reproductive canal, enabling it to carry out both of these functions [12,13,14,15]. Natural killer (NK) cells, macrophages, and neutrophils all gradually grow in number during the menstrual cycle, but they are most prevalent just before menstruation, which may indicate their function in the destruction of the endometrium and in host defense during the disruption of the mucosal barrier. Similar to this, adaptive immune cells, which are found in the endometrium as distinct aggregates made up of a B-cell core surrounded by T cells and an outside halo of macrophages, increase in number throughout the proliferative phase and momentarily lose their ability to cause cytotoxicity during the secretory phase, during which conception may take place. The extraordinary sensitivity of the immune system to hormonal changes in this specific milieu is shown by these findings [16,17,18].

Endometrial cancer has not received as much attention as other types of cancer regarding the composition of the tumor microenvironment (TME) and its relationship with prognosis. In contrast, the presence of intraepithelial tumor-infiltrating lymphocytes (TILs) has been established as a strong predictor of a more favorable prognosis in ovarian cancer, as demonstrated in a recent meta-analysis of ten trials [19,20,21,22]. However, early clinicopathologic investigations have yielded conflicting results regarding whether TILs are more abundant in low-grade endometrial malignancies compared to high-grade endometrial cancers or whether the location of TILs affects prognosis. Upon univariate analysis, perivascular lymphocytic infiltrates have been shown to be associated with low overall survival (OS), but multivariate analysis has revealed that intraepithelial lymphocytic infiltrates near the invasive boundary are associated with higher OS. Due to the variability of the research and the scarcity of available data, comparing studies and drawing significant conclusions are challenging.

Recent studies have focused on the ratio of CD8+ TILs to regulatory T cells (Tregs: CD4+ CD25+ FOXP3+), which are recognized for their physiological involvement in peripheral tolerance and their pathological role in antitumor immunity [23,24]. De Jong et al. found that the presence of many CD8+ TILs was an independent predictor of enhanced OS (total cohort and type II), and that a high CD8+/FoxP3+ ratio [25,26] was an independent predictor of increased disease-free survival (DFS) in type I, but not type II, endometrial cancer patients. These findings were obtained after adjusting for well-known prognostic variables in multivariate analysis. In another trial that did not differentiate by tumor type, the significance of the ratio of CD8+ to FoxP3+ T cells to DFS was validated. Although a statistically significant association has been shown between the presence of Tregs and tumor stage, grade, and the presence of myometrial invasion, the number of intra-tumoral Tregs alone has not been demonstrated to affect recurrence and survival curves. Myeloid cells, in contrast to lymphoid cells, appear to be more thoroughly understood regarding their involvement in endometrial disease. Although myeloid-derived suppressor cells (MDSCs) have been found in tumor tissues, tumor-associated macrophages (TAMs) have consistently been identified as major contributors to a pro-tumorigenic environment [27,28,29]. The progression of the disease from precancerous endometrial lesions (different types of hyperplasia) to endometrial cancer has been shown to lead to a steady increase in TAM density, particularly within the stromal compartment [30,31]. TAMs have frequently been associated with the more aggressive characteristics of the primary tumor, such as a higher stage and grade, as well as the presence of lymphovascular and myometrial invasion. Only one study’s findings, which were based on a small and diverse cohort of type I and type II carcinomas, deviated from these general conclusions. Additionally, pelvic lymph node metastases and an angiogenic character have both been substantially linked to the existence of TAMs.

Recently published data, such as those of Kubler et al., showed for the first time that TAM density is an independent prognostic factor for recurrence-free survival [29,32,33]. These authors found that a high density of TAMs compared to a low density increased the likelihood of recurrence by a factor of 8.3, which is a measure of prognosis. In their study, univariate analysis revealed a significant association between the presence of TAMs and overall survival, but the multivariate analysis did not reveal this relationship. This may also explain earlier research’s inability to detect anything more than a trend toward significance [34,35,36]. 

### 2.3. The Use of Immunotherapy for Endometrial Cancer

#### 2.3.1. Therapeutic Vaccination

**Figure 1:**
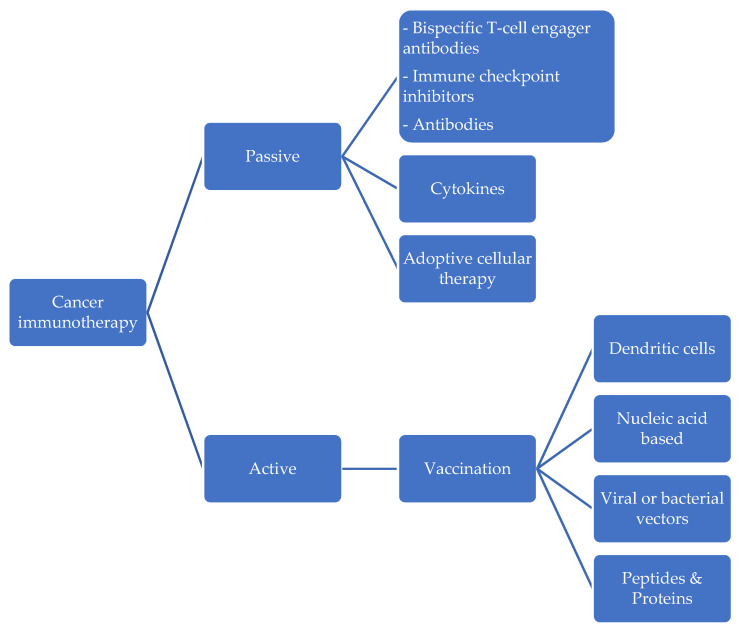
Immunotherapeutic approach to uterine cancer.

As presented in Figure 1, active immunotherapy takes the form of therapeutic cancer vaccination. Active immunotherapies encourage the host’s immune system to build an antitumor immune response and create immunological memory, potentially delivering a long-lasting impact even after the therapy is stopped. Cancer vaccines exploit the immune system’s cellular component by activating T lymphocytes to attack antigens linked with tumors (TAAs). In theory, cancer vaccines have the potential for high specificity, minimal toxicity, and sustained action, but these qualities have yet to be effectively translated into clinical practice [37]. The product of Wilms tumor gene 1 (WT1) has been determined to be a TAA with therapeutic promise for endometrial cancer. A transcription factor necessary for the healthy growth of the urogenital system is encoded by the gene WT1, which is found on chromosome 11p13. According to the immunohistochemical staining method, this gene has been found in anywhere between 0% and 79% of endometrial cancer cases [38].

A recent clinical trial in phase I evaluated the safety and tolerability of a WT1 peptide vaccination in 12 patients with recurrent or progressing gynecologic malignancies [39]. The vaccination consisted of a modified 9-mer WT1 peptide emulsified with Montanide ISA51 adjuvant and was restricted to the HLA-A2402 allele. The results showed that the vaccine was well-tolerated and safe for use in this patient population. Only erythema at the injection site was associated with adverse effects, and 25% of patients had stable disease (SD) or no illness at all during the first three months, compared to nine individuals who had progressive disease (PD). Cooseman et al. described the use of autologous dendritic cells loaded with WT1 mRNA as another method for the immunization of four patients with advanced serous endometrial cancer [40,41].

Regarding therapeutic manipulation, cancer testis (CT) antigens, which are exclusively expressed in male germ cells and placental tissue in healthy people but ectopically in the tumor cells of several human cancer types, have become attractive candidates. High tumor specificity and immunogenicity are made possible by the confined nature of their expression [42]. Endometrial cancer has so far been linked to several CT antigens. NYESO-1 and MAGE-A4 are present in 19% and 12% of endometrioid adenocarcinomas [43] and 32% and 63% percent of USC cases, respectively. Furthermore, SSX-4 and KU-CT-1 have been found in 24% and 64%, respectively, of endometrial cancer patients [44,45]. The safety and immunogenicity of recombinant vaccinia-NYESO-1 and recombinant fowlpox-NYESO-1 were tested in a two-part, open-label cohort study, comprising 36 patients with a variety of tumor types. The reactions to the vaccination, which included erythema and pruritis at the injection site, were comparable but not severe, although the immunologic responses of the patients varied greatly [46]. The transmembrane receptor human epidermal growth factor receptor 2 (HER-2/neu), which is encoded by the ERBB2 gene, also has promise for the treatment of endometrial cancer. The overexpression of HER2/neu is related to lower overall survival in USC in particular, varying from 16% to 80% [47].

#### 2.3.2. Options for Immunotherapeutics

The endometrial immune system has unusual physiological characteristics because it has a dual job: on the one hand, it should defend against infections and sexually transmitted pathogens, but on the other hand, it should facilitate the implantation of an allogenic embryo. Sex hormones acting through the menstrual cycle impact the endometrial microenvironment to carry out these functions.

Immunotherapy, which appears to be the future of cancer treatments, mainly entails triggering the body’s own immune response, particularly against tumor cells. Several drugs are available that target various biological pathways. Some of these medications have previously been licensed for the treatment of non-gynecological cancers including lung cancer and melanoma, and they may also be very effective in the management of EC. These treatments are divided into two categories: active therapy and passive therapy. The former is based on the injection of exogenously created or modified immune system components stimulating an anti-tumor immune response, while the former stimulates the host’s own immune system against cancer cells.

#### 2.3.3. Adoptive Cellular Therapy

In the context of passive immunotherapy, adoptive cellular therapy antibodies, cytokines, and lymphocytes created or modified exogenously are administered to encourage an immune response that fights tumors. They provide immediate but transient immunity, since they cannot create immunological memory. Cells from the blood or bone marrow are extracted, activated, and grown in vitro before being reinfused into the same patient (autologous) or a new patient in adoptive cellular therapy (allogeneic). Significant advancements in technology have made possible the creation of genetically altered tumor-reactive T cells that express recombinant or chimeric T-cell receptors specific for common TAAs (CAR T cells).

The current strategies for inducing tumor cell death involve immune system activation. However, immunotherapy has recently shifted towards blocking the inhibitors that prevent an effective immune response. This is because the evasion of the immune system by tumor cells diminishes the efficacy of both active and passive immunotherapies. Among the newer approaches to counter immune tolerance, immunological checkpoint inhibitors are the most promising. 

Several immune checkpoint inhibitors have been approved for the treatment of various types of cancer, including uterine cancer. The most widely studied immune checkpoint inhibitors are pembrolizumab and nivolumab, which target the PD-1 receptor of T cells.

In a phase II clinical trial, pembrolizumab was found to be effective in the treatment of recurrent or metastatic uterine cancer [48]. The overall response rate was 13.3%, with a median response duration of 10.3 months. In another phase II trial, nivolumab was found to have an overall response rate of 20% in patients with advanced or recurrent uterine cancer [49].

However, not all patients with uterine cancer respond to immune checkpoint inhibitors, and no biomarkers that can reliably predict response to treatment exist currently. One study found that uterine cancer patients with high levels of the immune checkpoint protein LAG-3 had a poorer response to pembrolizumab [50]. Other potential biomarkers for response to immune checkpoint inhibitors in uterine cancer include tumor mutational burden, PD-L1 expression, and tumor-infiltrating lymphocytes [51].

Combination therapy with immune checkpoint inhibitors and other cancer treatments, such as chemotherapy, radiation therapy, or other immunotherapies, are also being investigated for the treatment of uterine cancer. A phase II clinical trial found that the combination of pembrolizumab and chemotherapy had a higher response rate (22.5%) than pembrolizumab alone [52].

Dostarlimab is a monoclonal antibody that targets the PD-1 receptor on T cells and is approved for the treatment of certain cancers, including endometrial cancer, which is a type of uterine cancer. Dostarlimab works by blocking the interaction between PD-1 and its ligands, PD-L1 and PD-L2, which allows T cells to recognize and attack cancer cells. In a phase I clinical trial, dostarlimab was found to be safe and effective in the treatment of recurrent or advanced endometrial cancer [53]. The overall response rate was 30%, with a median response duration of 25.3 months. These results led to the approval of dostarlimab for the treatment of recurrent or advanced endometrial cancer in April 2021 [54].

Dostarlimab is also being investigated in combination with other cancer treatments, including chemotherapy and radiation therapy, for the treatment of uterine cancer. In a phase III clinical trial, the combination of dostarlimab and chemotherapy was found to be more effective than chemotherapy alone in patients with advanced or recurrent endometrial cancer [55].

However, as with other immunotherapies, not all patients with uterine cancer respond to dostarlimab, and biomarkers for identifying patients who are most likely to benefit from treatment are needed. One potential biomarker for response to dostarlimab in endometrial cancer is microsatellite instability (MSI), which is a type of genomic instability that occurs in about 20% of endometrial cancers [56]. In a phase II clinical trial, patients with high-MSI endometrial cancer had a higher response rate to dostarlimab than patients with stable-MSI endometrial cancer [57].

A recent study showed that the combination of lenvatinib and pembrolizumab was more effective than standard chemotherapy in the treatment of advanced endometrial cancer [58]. The study was a phase III clinical trial that included patients with recurrent or metastatic endometrial cancer who had previously received at least one line of systemic therapy. The trial compared the combination of lenvatinib, an oral tyrosine kinase inhibitor, and pembrolizumab, a monoclonal antibody that targets the programmed death-1 (PD-1) receptor, with standard chemotherapy. The study found that patients who received lenvatinib plus pembrolizumab had longer median progression-free survival compared to those who received chemotherapy (6.6 vs. 3.8 months in the pMMR population, and 7.2 vs. 3.8 months overall). The combination therapy also resulted in longer median overall survival compared to chemotherapy (17.4 vs. 12.0 months in the pMMR population, and 18.3 vs. 11.4 months overall). Despite the promising results, the combination therapy was associated with higher rates of adverse events of grade 3 or higher compared to chemotherapy (88.9% vs. 72.7%, respectively). However, most adverse events were manageable with dose modifications or interruptions, and the discontinuation rate due to adverse events was similar between the two groups. These findings suggest that the combination of lenvatinib and pembrolizumab could be an effective treatment option for patients with advanced endometrial cancer who have progressed under previous therapies. Further research is needed to determine the optimal treatment sequence and to identify predictive biomarkers for response to this therapy [58].

**Figure 2:**
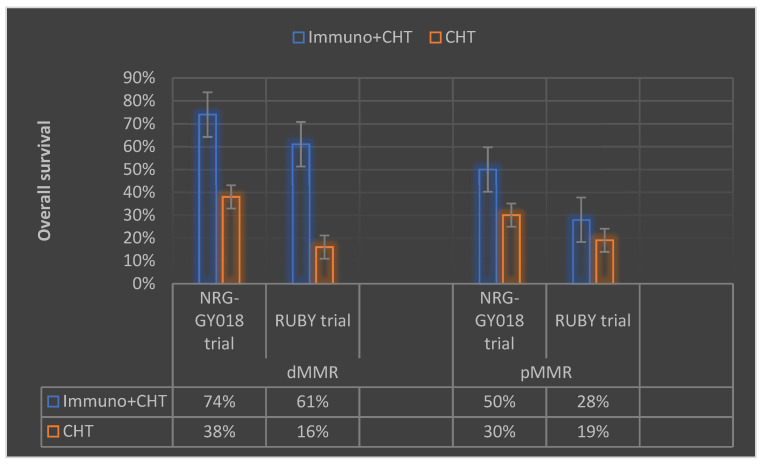
Summary of the recently reported data from the trials NRG-GY018 and RUBY on endometrial cancer immunotherapy presented at the 2023 Society of Gynecologic Oncology (SGO) Annual Meeting (Immuno—immunotherapy, CHT—chemotherapy, dMMR—deficient mismatch repair, pMMR—proficient mismatch repair).

According to a recent report presented at the Society of Gynecologic Oncology (SGO) Annual Meeting, adding immunotherapy to chemotherapy remains the standard treatment approach for endometrial cancer patients, regardless of disease stage (Figure 2). In recent trials, the phase III NRG-GY018 [59] and phase III RUBY [60] studies have reported positive results in endometrial cancer subgroups. 

The NRG-GY018 study of pembrolizumab showed a statistically significant and clinically meaningful improvement in progression-free survival in patients with stage III-IV or recurrent endometrial carcinoma, regardless of mismatch repair status. The RUBY study investigated dostarlimab in patients with dMMR and high-microsatellite-instability tumors, demonstrating a significant PFS improvement compared to the placebo group. The FDA has yet to approve these studies, but many expect these agents to change clinical practice and be incorporated into frontline and recurrent settings. However, treatment with these agents is associated with adverse events, and the safety profile requires further investigation.

### 2.4. Future Perspectives

Immunotherapy in uterine cancer holds great promise, necessitating further research and clinical trials to optimize its use and identify novel targets [61]. Combination strategies involving immune checkpoint inhibitors and other treatment modalities such as chemotherapy, radiation therapy, and targeted therapies have the potential to enhance response rates and improve patient outcomes [49]. Ongoing efforts to unravel the intricate tumor microenvironment and immune escape mechanisms in uterine cancer are expected to provide crucial insights into novel therapeutic targets [62]. Personalized immunotherapeutic approaches, tailored to the unique molecular and immunological profiles of individual patients, represent an exciting avenue for exploration [63]. This individualized approach has the potential to enhance treatment efficacy and mitigate immune-related adverse events, as demonstrated in studies investigating immune checkpoint inhibitor therapy in rheumatic diseases [64] Advancements in technologies such as neoantigen identification, adoptive cell therapies, and immune monitoring techniques have the potential to revolutionize the field [65]. Interdisciplinary collaborations among clinicians, immunologists, molecular biologists, and computational scientists are essential to accelerating the translation of immunotherapeutic breakthroughs into clinical practice, ultimately offering new hope and improved outcomes for patients with uterine cancer [66].

## 3. Conclusions

Immunotherapy has emerged as a promising treatment option for uterine cancer, and ongoing studies are focused on enhancing clinical responses through immunotherapy techniques. While endometrial cancer has been shown to be immunogenic enough for immunomodulation, efforts to increase the role of active and/or passive immunotherapy in treating the disease have been limited. This is concerning, since endometrial cancer is the only gynecologic cancer with an increasing incidence and death rate, making the identification of effective treatments crucial for patients with metastatic or recurring illnesses. Small preclinical and phase I trials evaluating the effectiveness of immunotherapy in such patients have shown positive immunologic responses but limited clinical responses, leading to concerns about proving therapeutic efficacy in a small group of extensively pretreated patients with advanced illness and short follow-up. The key challenges in determining the effectiveness of immunotherapy for endometrial cancer are identifying the subgroup of patients most likely to respond to immunotherapy, identifying biomarkers that can predict treatment success, and finding therapeutic combinations that can improve medication performance while reducing toxicity.

## Data Availability

The data presented in this study are available upon request from the corresponding author.
